# TRUS-Guided Target Biopsy for a PI-RADS 3–5 Index Lesion to Reduce Gleason Score Underestimation: A Propensity Score Matching Analysis

**DOI:** 10.3389/fonc.2021.824204

**Published:** 2022-01-24

**Authors:** Jae Hoon Chung, Byung Kwan Park, Wan Song, Minyong Kang, Hyun Hwan Sung, Hwang Gyun Jeon, Byong Chang Jeong, Seong Il Seo, Seong Soo Jeon, Hyun Moo Lee

**Affiliations:** ^1^ Department of Urology, Samsung Medical Center, Sungkyunkwan University School of Medicine, Seoul, South Korea; ^2^ Department of Radiology, Samsung Medical Center, Sungkyunkwan University School of Medicine, Seoul, South Korea

**Keywords:** prostatic neoplasms, biopsy, Gleason score, prostate imaging and reporting and data system, transrectal ultrasound

## Abstract

**Background:**

Magnetic resonance imaging (MRI) and transrectal ultrasound (TRUS)-guided cognitive or image fusion biopsy is performed to target a prostate imaging reporting and data system (PI-RADS) 3–5 lesion. Biopsy Gleason score (GS) is frequently underestimated compared to prostatectomy GS. However, it is still unclear about how many cores on target are necessary to reduce undergrading and if additional cores around the target may improve grade prediction on surgical specimen.

**Purpose:**

To determine the number of target cores and targeting strategy to reduce GS underestimation.

**Materials and Methods:**

Between May 2017 and April 2020, a total of 385 patients undergoing target cognitive or image fusion biopsy of PI-RADS 3–5 index lesions and radical prostatectomies (RP) were 2:1 matched with propensity score using multiple variables and divided into the 1–4 core (*n* = 242) and 5–6 core (*n* = 143) groups, which were obtained with multiple logistic regression with restricted cubic spline curve. Target cores of 1–3 and 4–6 were sampled from central and peripheral areas, respectively. Pathologic outcomes and target cores were retrospectively assessed to analyze the GS difference or changes between biopsy and RP with Wilcoxon signed-rank test.

**Results:**

The median of target cores was 3 and 6 in the 1–4 core and 5–6 core groups, respectively (*p <* 0.001). Restricted cubic spline curve showed that GS upgrade was significantly reduced from the 5th core and there was no difference between 5th and 6th cores. Among the matched patients, 35.4% (136/385; 95% confidence interval, 0.305–0.403) had a GS upgrade after RP. The GS upgrades in the 1–4 core and 5–6 core groups were observed in 40.6% (98/242, 0.343–0.470) and 26.6% (38/143, 0.195–0.346), respectively (*p =* 0.023). Although there was no statistical difference between the matched groups in terms of RP GS (*p =* 0.092), the 5–6 core group had significantly higher biopsy GS (*p =* 0.006) and lower GS change from biopsy to RP (*p =* 0.027).

**Conclusion:**

Five or more target cores sampling from both periphery and center of an index tumor contribute to reduce GS upgrade.

## Introduction

Gleason score (GS) can be used to assess the aggressiveness and prognosis of prostate cancer (PCa) (1). However, pathologic discrepancies between pre-operative biopsy and radical prostatectomy (RP) in terms of GS are common (1, 2). GS underestimation is reported to range from 19% to 57% (3–9). The possibility of GS upgrade after RP compared with that after prostate biopsy is well known (10). The low-, intermediate-, and high-risk categories are based on Gleason score as this categorization drives treatment planning. Underestimating Gleason 8 (high risk) as Gleason 6 (low risk) has more clinical impact than underestimating Gleason 10 (high risk) as a Gleason 8 (high risk) (11). Moreover, incorrect GS biopsy can adversely impact treatment for men with PCa (12).

Recently, there has been an increase in usage of multi-parametric magnetic resonance imaging (mpMRI) for prostate-targeted biopsy owing to its significant accuracy for pre-operative PCa diagnosis (13, 14). Many studies reported that mpMRI-targeted biopsy of suspected lesions increases detection of clinically significant PCa (15, 16). Calio et al. reported that saturation target biopsy of an index lesion significantly decreases the risk of upgrading on radical prostatectomy by minimizing the impact of tumor heterogeneity (17). However, there is no consensus on the number of target biopsy cores required per lesion to minimize underestimation of GS during mpMRI-targeted biopsy. Additionally, it is unclear if central sampling of an index tumor is the best strategy for target biopsy. Many radiologists and urologists try to target the center of an index tumor alone. However, this targeting can make it difficult to detect additional significant cancers in the peripheral area of an index tumor, in which tissue heterogeneity is frequent in high GS cancer (17).

However, only a few reports have focused on the strategy of target biopsy and the number of target cores that contribute to reducing GS underestimation (18–22). If prostate biopsy can predict or represent an RP specimen, it would be helpful to determine treatment options and predict prognosis. This study hypothesized that sampling the peripheral and central areas of an index tumor improves GS discrepancies between biopsy and prostatectomy, and that the GS discrepancy is changed according the number of target cores. The purpose of this study was to assess our strategy for target biopsy and to determine the number of target cores to reduce GS underestimation.

## Materials and Methods

This study was performed in agreement with the applicable laws and regulations, good clinical practices, and ethical principles as described in the Declaration of Helsinki. Our institutional review boards approved the present study (2020-08-137) and waived the need for informed consent.

### Patients

**Figure 1:**
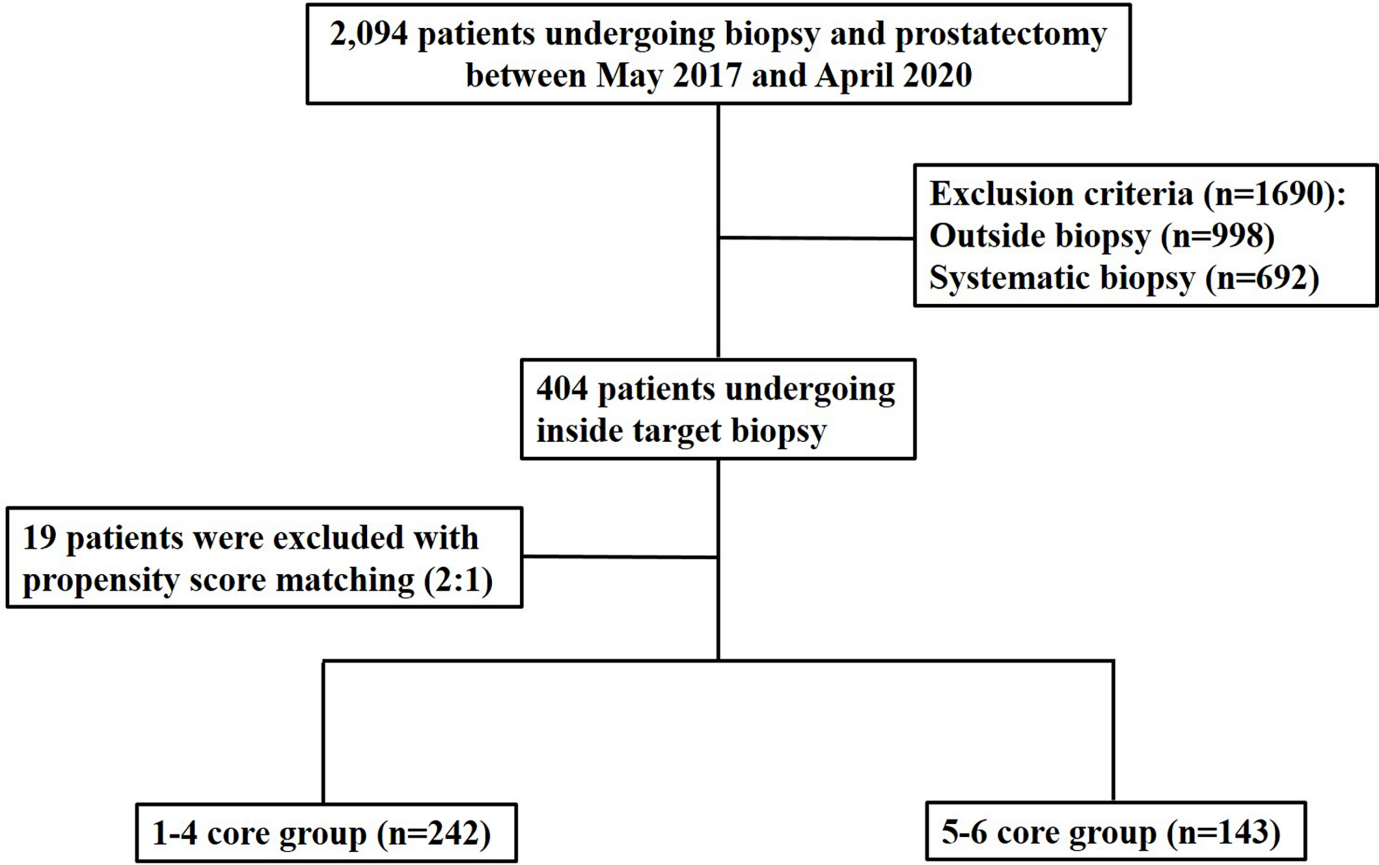
Flow diagram illustrating how to include and to exclude patients.

We reviewed a total of 2,094 patients who underwent RP between May 2017 and April 2020. Among them, we included patients who were diagnosed with PCa through mpMRI target biopsy by two genitourinary radiologists. Both radiologists were experts in mpMRI target biopsies and had performed more than 250 prostate biopsies per year. Among the 2,094 patients, 998 were excluded because PCa was diagnosed at other institutions. Of the remaining 1,096 patients, 692 were excluded because PCa was diagnosed by systematic biopsy alone. Finally, a total of 404 patients were included in the analysis (**Figure 1**). Of these patients, 22.8% (92/404) had a history of past biopsies. Prostate biopsies were performed in the radiologic department of a single institute. The median time interval between biopsy and RP was 59.0 days [45–81 days]. Pathological reporting was assessed by one genitourinary pathologist who had specialized in genitourinary pathology for 20 years. He examined both biopsy and RP specimens to determine GS, tumor size, and cancer stage based on the 2014 International Society of Urological Pathology Consensus Conference (23). We defined GS upgrade when biopsy GS was upgraded into prostatectomy GS by 1 or more and when biopsy GS 7 (3 + 4) was changed into prostatectomy GS 7 (4 + 3). The entire RP specimen was multi-sectioned, but giant micro-slides that covered both lobes were not created.

### Clinico-Pathological Parameters

Baseline characteristics of age at RP, body mass index (BMI), prostate-specific antigen (PSA) level before prostate biopsy, prostate volume (measured on MRI), PSA density (PSAD), alpha-reductase inhibitor, and history of prostate biopsy were evaluated. Pathologic outcomes and number of biopsy cores were compared to analyze the difference between biopsy and RP or GS changes from biopsy to RP. Biopsy complications were recorded to assess whether increasing the number of target biopsy cores influenced post-biopsy complication rates.

### MRI Protocol and Interpretation

All MRI examinations were performed with a 3-T scanner (Intera Achieva TX, Philips Healthcare, Best, Netherlands) using a phase-array coil (Philips Healthcare). Standard MRI parameters for both prostate imaging reporting and data system (PI-RADS) versions 2.0 were applied. MR images were interpreted and prostate biopsies were performed by one of two radiologists. MRI protocols included T2-weighted imaging (T2WI), T1-weighted imaging, diffusion-weighted imaging (DWI), apparent diffusion coefficient (ADC) imaging, and dynamic contrast-enhanced imaging (DCEI). The T2WI was scanned into axial, sagittal, or coronal planes. The other MR sequences were scanned into only the axial plane. DWI was scanned with *b*-values of 0, 100, 1000, and 1500 s/mm^2^. ADC values were calculated with all *b*-values for the DWI, and ADC map images were created. DCEI was obtained with an ultra-fast scan that covered the entire prostate.

Interpretation of MR images was based on PI-RADS version 2.0. Tumor size of a peripheral index lesion was measured on DWI, and that of a transition index lesion was measured on T2WI. Additionally, the size of an index tumor was also measured on the RP specimen to compare between target core groups.

### mpMRI-Target Biopsy

Target biopsy (median, 4.00 cores and range, 1–6) was performed on the index tumor, which was categorized as PI-RAD 3–5. Cognitive to image fusion biopsy ratio was 156:248. One of two radiologists performed image fusion biopsy in 156 patients and cognitive biopsy in 29 patients. He obtained mainly three or less target cores from the center of an index lesion. The other performed only cognitive biopsy in 219 patients and he obtained mainly 6 or less target cores from the central and peripheral areas of an index lesion.

**Figure 2:**
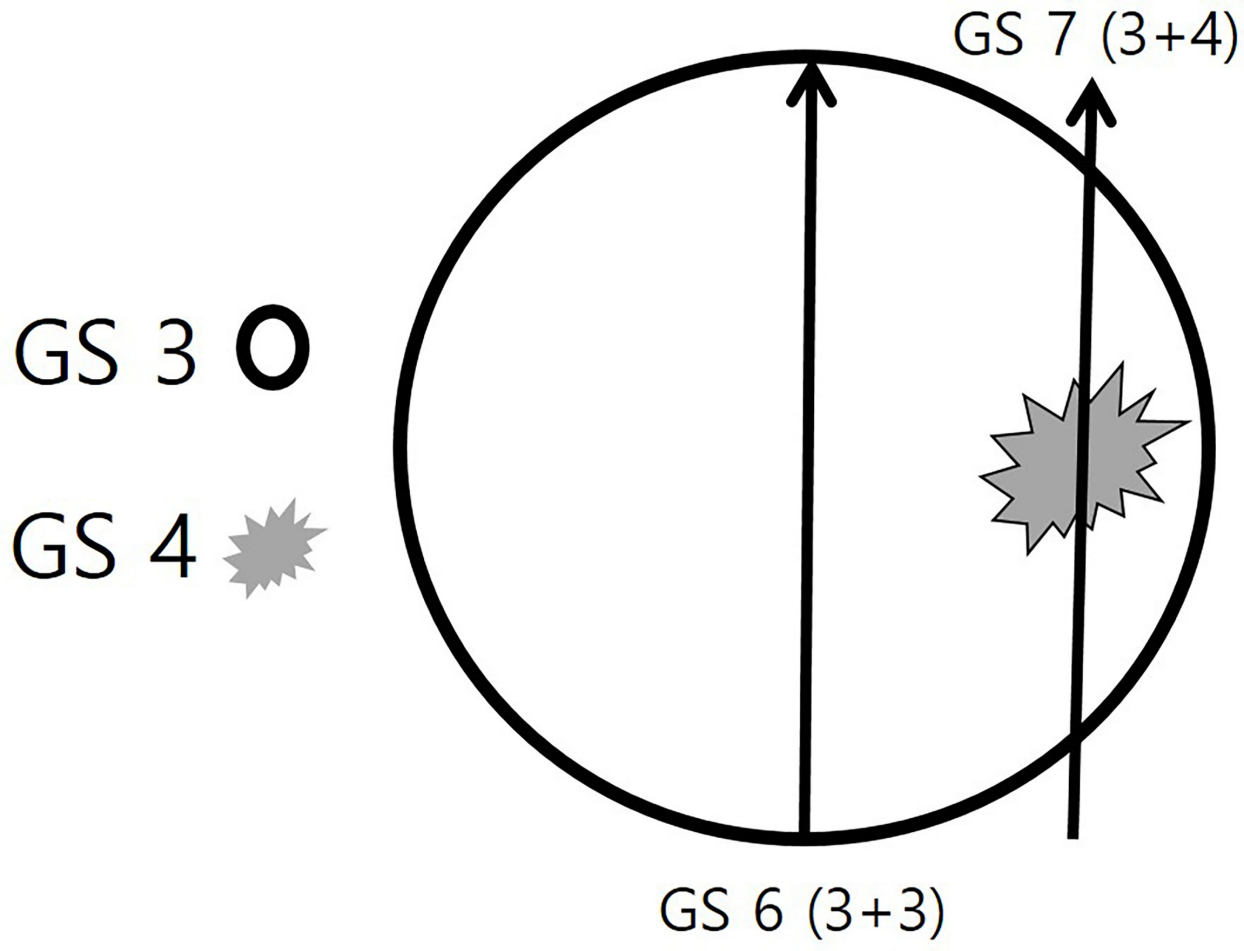
Schematic illustration of the target biopsy strategy. A round prostate cancer consists of two Gleason score (GS) components of GS 3 and GS 4. The GS 3 (black circle) is the major component and the GS 4 (gray spiculation) is the minor component. If a radiologist or urologist targets the central area alone, the histologic diagnosis will be a GS 6 (3 + 3) adenocarcinoma. However, if the periphral area is targeted, the histologic diagnosis will be a GS 7 (3 + 4) adenocarcinoma.

The first, second, and third target cores were sampled in the center of an index lesion, and the other (fourth, fifth, and sixth) target cores were sampled in the peripheral area of the index tumor (**Figure 2**). Central sampling was followed by peripheral sampling for target biopsies. Accordingly, number 1–3 target cores indicated central sampling, and number 4–6 target cores indicated peripheral sampling (24). Two radiologists used one of three US scanners including EPIC (Philips Health Care, Bothell, WA, USA), IU22 (Philips Health Care), or Aplio 500 (Toshiba Medical System, Japan). End-fire US probe was introduced into the rectum. Fly Thru and Smart Fusion (Toshiba Medical System) was used for MRI-TRUS fusion imaging. An 18-gauge needle mounted on a spring-loaded commercial biopsy device (ACECUT; TSK Laboratory, Tochigi-shi, Japan) was used for target and systematic biopsies.

### Statistical Analysis

Univariate and multivariate analyses were performed to identify what influenced the GS upgrade. Multiple logistic regression was used to determine if there was GS upgrade according to the number of biopsy cores. Also, restricted cubic spline curve was generated to show linearity of the GS upgrade rate as the number of cores increases. It was significantly reduced from the 5th core, but there was no difference between the 5th and 6th cores. Accordingly, we divided the study population into the 1–4 core group and 5–6 core group.

Standardized mean difference (SMD) was assessed to detect how baseline characteristics were changed before and after matching, The cases were matched with 2:1 propensity score using these variables to compare 1–4 core and 5–6 core groups in terms of GS upgrade, GS difference or change, and complication.

Age and BMI, in which these data were in normal distribution, were compared between the groups using *t*-test. Wilcoxon rank sum test was used to compare the other variables in baseline characteristics because these data were not in normal distribution. This statistical test was used to compare GS difference or change from biopsy to RP. Chi-square test was also used to compare percentage data between groups. Data were shown as mean ± standard deviation or median [interquartile range]. Statistical analyses were performed with R version 4.1.0 (2021-05-18) (Vienna, Austria; http://www.R-project.org/). All two-sided *p*-values < 0.05 were considered statistically significant.

## Results

Univariate analysis showed that PI-RADS score (*p =* 0.027–0.003) and number of target cores (*p =* 0.006) significantly influenced GS upgrade (**Table 1**). Multivariate analysis demonstrated that these variables were also involved in GS upgrade (**Table 1**). The *p*-values of PI-RADS score and number of target cores were 0.043–0.007 and 0.012, respectively. The odds ratios of these variables ranged from 0.396 to 0.533 (**Table 1**) and thus GS upgrade decreased 0.396–0.533 times as the PI-RADS scores and the number of target cores increased. The other variables such as age, body mass index, previous biopsy history, 5α-reductase inhibitor, PSA, prostate volume, PSAD, and the size of an index lesion did not significantly influence GS upgrade (*p =* 0.169-0.981).

**Table 1:** Clinical variables influencing GS upgrade with univariate and multivariate analyses.

Clinical variables	Simple logistic regression						Multiple logistic regression			
	Odds ratio	Lower limit			Upper limit	*p*-value	Odds ratio	Lower limit	Upper limit	*p*-value
Age	0.996	0.965			1.028	0.807				
Body mass index	1.042	0.966			1.124	0.291				
Number of previous biopsies										
0	Reference									
1	0.694	0.413			1.168	0.169				
2 and 3	1.250	0.275			5.684	0.7727				
Alpha reductase inhibitor	1.482		0.742	2.959		0.265				
Prostate-specific antigen	1.002		0.973	1.032		0.903				
Prostate volume	0.994		0.980	1.009		0.418				
Prostate specific antigen density	1.042		0.442	2.455		0.925				
Size of index lesion	0.957		0.702	1.304		0.781				
PI-RADS										
3	Reference									
4	0.368		0.189	0.714		0.003	0.396	0.202	0.776	0.007
5	0.456		0.227	0.916		0.027	0.483	0.239	0.979	0.043
Number of target cores	0.832		0.729	0.949		0.006	0.533	0.326	0.870	0.012

PI-RADS, prostate imaging and reporting and data system.

**Figure 3:**
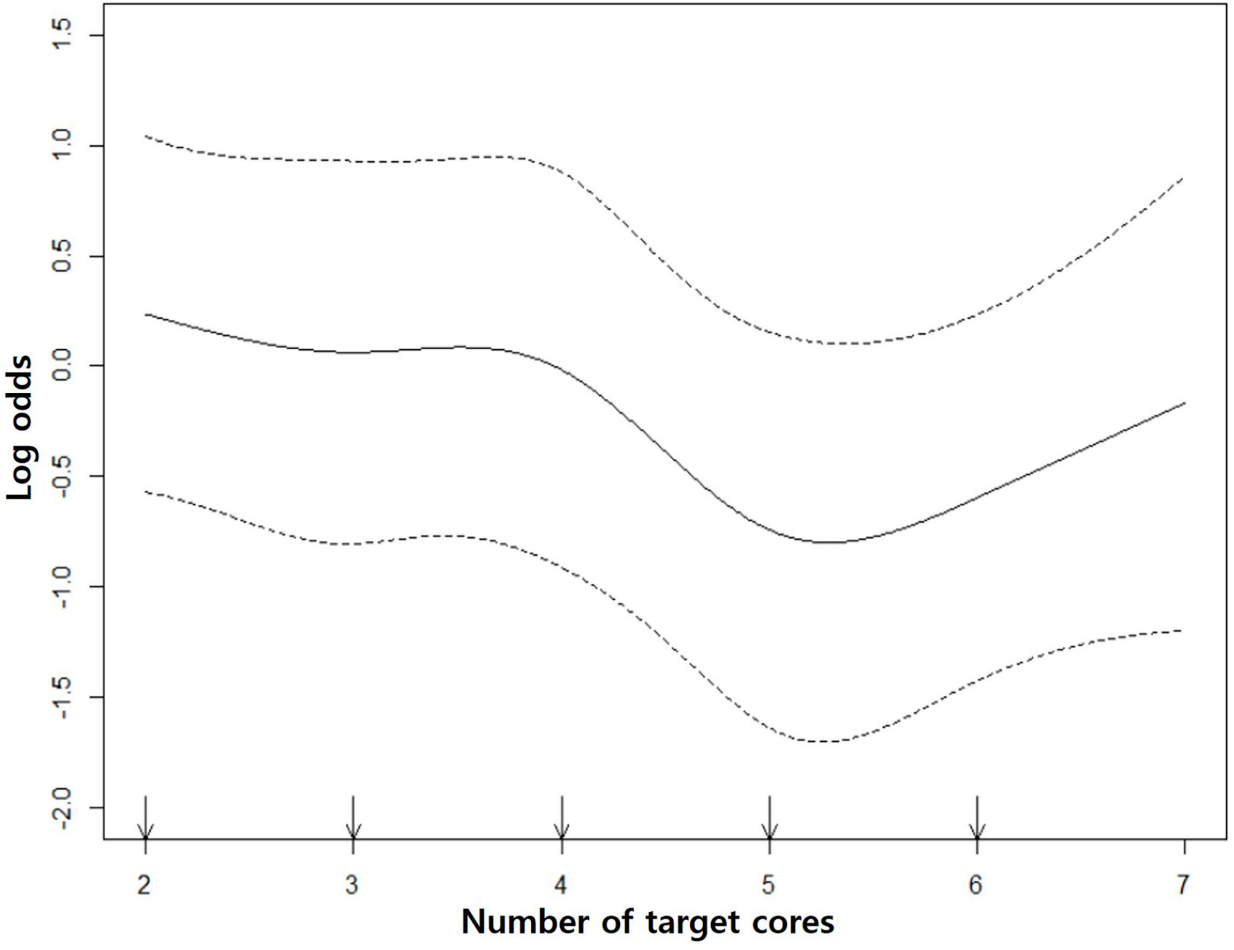
Restricted cubic spline curves. The middle solid curve shows the change of GS upgrade according to the number of target cores. It decreases from the 5th core and there is no significant GS change between 5th and 6th cores. The upper and lower dotted curves indicate the upper and lower limits of log odds.

Spline curve showed that GS upgrade was significantly reduced from the 5th core. However, the decreasing rate of the GS upgrade did not tend to have linearity as the number of target cores increased. Therefore, our study population was divided into the 1–4 core group (*n* = 260) and the 5–6 core group (*n* = 144) (**Figure 3**).

**Figure 4:**
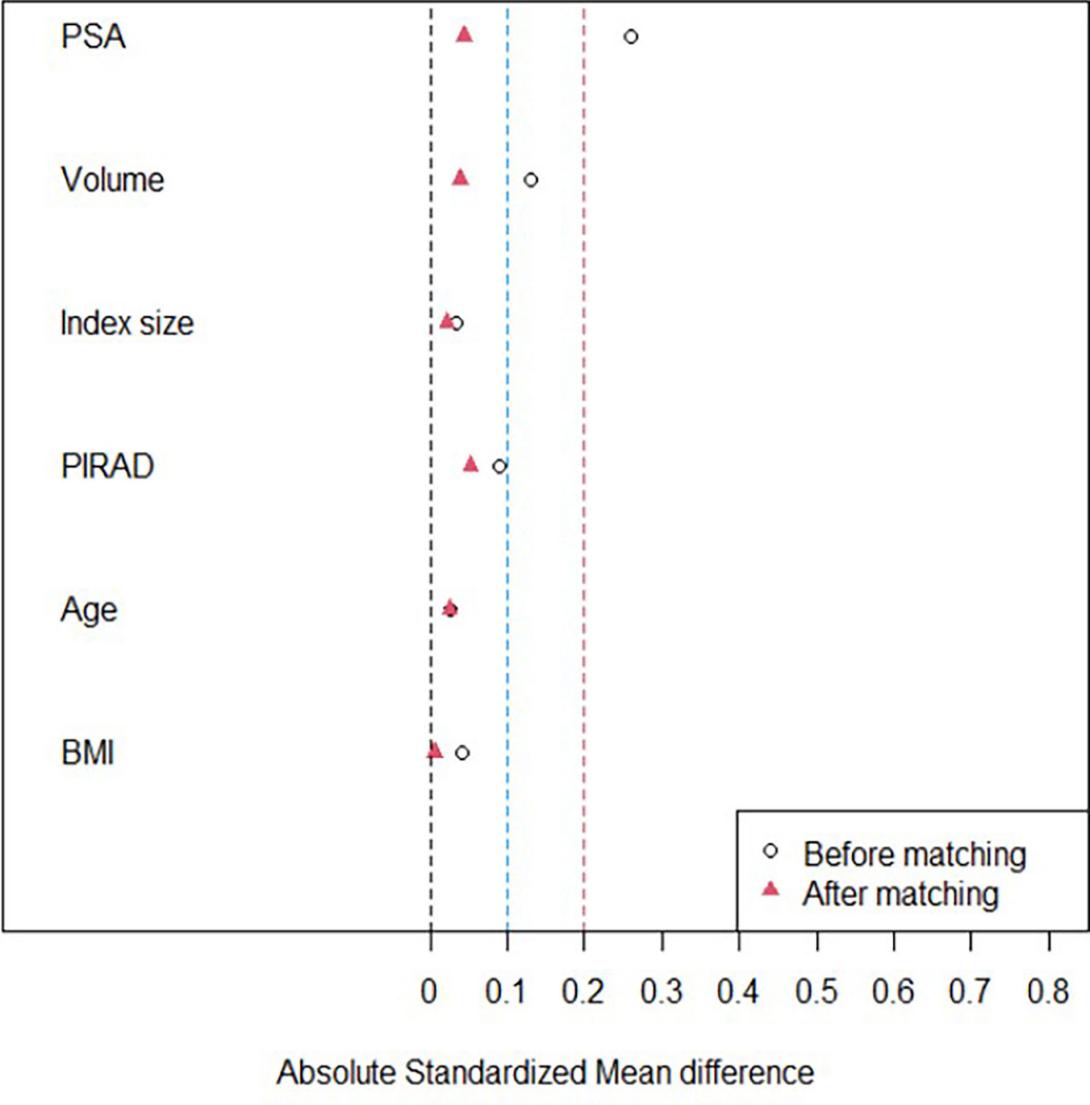
A graph illustrating the change of standardized mean difference (SMD). The SMDs of PSA, prostate volume, and PI-RADS scores are significantly reduced to 0.1 or less after propensity score matching. However, the SMDs of other baseline characteristics such as age, body mass index (BMI), and so on are not siginifcantly different before and after matching.

However, there was significant difference between the groups in terms of PSA (*p =* 0.038) (**Table 2**). The SMDs of PSA, prostate volume, and PI-RADS score were significantly reduced from 0.26 to 0.04, from 0.13 to 0.04, and from 0.1 to 0.05 after matching, respectively (**Figure 4**) (**Table 2**). However, the SMDs of baseline characteristics such as age and BMI were not so different after matching (**Figure 4**) (**Table 2**). Therefore, the cases were matched with 2:1 propensity score using PSA, prostate volume, PI-RADS score, age, and BMI (**Table 2**).

**Table 2:** Baseline characteristics before and after propensity score matching.

Pre-biopsy variables	Before matching				After matching			
	1–4 core group	5–6 core group	*p*-value	SMD	1–4 core group	5–6 core group	*p*-value	SMD
No. of pts	260	144			242	143		
Age (years)	65.99 ± 6.44	65.83 ± 6.63	0.806	0.03	65.66 ± 6.45	65.83 ± 6.65	0.814	0.03
BMI	25.15 ± 2.69	25.04 ± 2.72	0.696	0.04	24.99 ± 2.69	25.01 ± 2.70	0.952	0.01
PSA	5.66 [4.04, 8.48]	4.92 [3.86, 7.09]	0.038	0.26	5.31 [3.82, 7.36]	4.88 [3.83, 7.09]	0.433	0.04
Volume	30.10 [24.60, 39.12]	29.55 [24.05, 37.58]	0.463	0.13	30.00 [24.45, 38.10]	29.55 [24.05, 37.58]	0.838	0.04
PSAD	0.17 [0.12, 0.29]	0.17 [0.12, 0.24]	0.398	0.18	0.17 [0.12, 0.24]	0.17 [0.12, 0.24]	0.873	0.01
Index size	1.20 [0.85, 1.70]	1.20 [0.90, 1.60]	0.718	0.04	1.18 [0.85, 1.60]	1.20 [0.90, 1.60]	0.473	0.02
PI-RADS			0.688	0.1			0.871	0.05
3 (%)	29 (11.2)	14 (9.7)			27.1 (11.2)	14.0 (9.8)		
4 (%)	142 (54.6)	85 (59.0)			138.8 (57.3)	85.0 (59.4)		
5 (%)	89 (34.2)	45 (31.2)			76.2 (31.5)	44.0 (30.8)		

[] indicates interquartile range. SMD, standardized mean difference; No. of pts, number of patients; BMI, body mass index (kg/m^2^); PSA, prostate-specific antigen (ng/ml); Volume, prostate volume (ml); PSAD, prostate-specific antigen density (ng/ml^2^); Index size, the size of an index lesion (cm); PI-RADS, prostate imaging and reporting and data system.

The number of study population was reduced from 404 to 285 because 19 patients were excluded due to the matching (**Figures 1** and **2**). The numbers of the 1–4 core and 5–6 core groups were reduced to 242 and 143, respectively (**Table 2**). PSA, which was significantly different between the 1–4 core and 5–6 core groups before the matching (*p =* 0.038), was not significantly different between the groups after the matching (*p =* 0.433) (**Table 2**). The other variables in baseline characteristics were not significantly different between the matched groups before (*p =* 0.398–0.806) and after (*p =* 0.433–0.952) the matching (**Table 2**).

**Figure 5:**
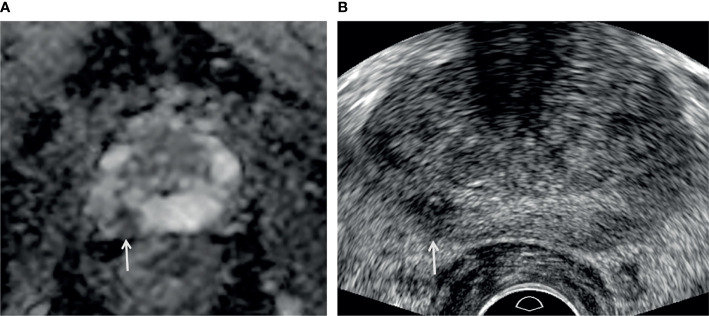
A 73-year-old man with high PSA (3.44 ng/ml). **(A)** Apparent diffusion coefficient map axial image (*b* = 1,500 s/mm^2^) shows a PI-RADS 4 lesion (white arrow) in the right peripheral apex. **(B)** Transrectal-ultrasound transverse image shows a hypoechoic mass (white arrow) well correlated with that on MRI. Target cores of 1–3 and 4–6 were sampled from the center and periphery of the index tumor, respectively. Target cores of 1–4 and 6 were confirmed as GS 6 (3 + 3) adenocarcinoma, but a target core of 5 was GS 7 (3 + 4) adenocarcinoma. A radical prostatectomy specimen demonstrated a final diagnosis of GS 7 (3 + 4) adenocarcinoma.

Overall GS upgrade was detected in 35.4% (136/385; 95% confidence interval, 0.305–0.403) in all matched patients. The GS upgrade of the 1–4 core and 5–6 core groups was 40.6% (98/242, 0.343–0.470) and 26.6% (38/143, 0.195–0.346) (**Figure 5**) (**Table 3**). However, no GS change or GS downgrade was not different between the matched groups.

**Table 3:** Biopsy and prostatectomy findings before and after propensity score matching.

Post-biopsy variables	Before matching			After matching		
	1–4 core group	5–6 core group	*p*-value	1–4 core group	5–6 core group	*p*-value
	(*n* = 260)	(*n* = 144)		(*n* = 242)	(*n* = 143)	
Positive in DRE	28 (10.7)	13 (9.0)	0.611	24 (9.9)	13 (9.1)	0.859
Previous biopsy	0.25 ± 0.48	0.24 ± 0.49	0.828	0.25 ± 0.48	0.24 ± 0.49	0.914
Biopsy naïve	200 (76.9)	112 (77.8)	0.844	187 (77.3)	111 (77.6)	0.937
Number of target cores	3 [2, 3.25]	6 [5, 6]	<.001	3 [2, 4]	6 [5, 6]	<.001
Detection rate^*^	79.66 ± 35.33	80.73 ± 27.68	0.755	78.98 ± 35.51	80.59 ± 27.73	0.641
Percentage of malignant tissue within biopsy cores	50 [22.5, 70]	60 [40, 72.5]	0.063	50 [20, 70]	60 [40, 70]	0.019
Biopsy GS	7 [6, 7]	7 [7, 7]	0.008	7 [6, 7]	7 [6.75, 7]	0.006
Prostatectomy GS	7 [7, 7]	7 [7, 7]	0.207	7 [7, 7]	7 [7, 7]	0.092
GS change (%)			0.010			0.023
No change	116 (44.6)	84 (58.3)		113.4 (46.9)	84.0 (58.7)	
Upgrade	107 (41.2)	38 (26.4)		98.2 (40.6)	38.0 (26.6)	
Downgrade	37 (14.2)	22 (15.3)		30.5 (12.6)	21.0 (14.7)	
Complication (%)	13 (5.0)	4 (2.8)	0.420	12.7 (5.2)	4.0 (2.8)	0.196

[] indicates interquartile range. GS, Gleason score. ^*^number of positive cores/total target cores.

**Figure 6:**
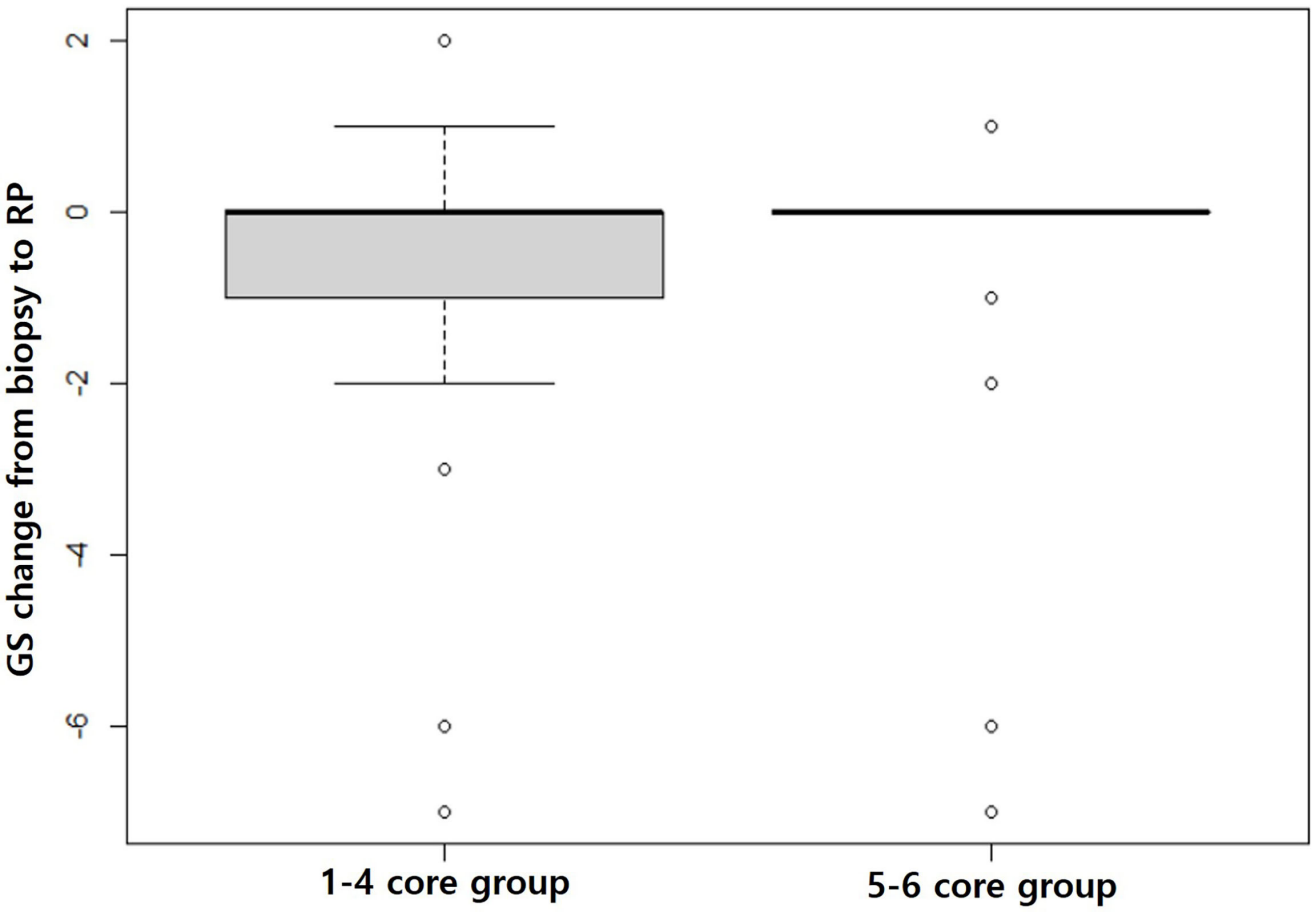
Box plots showing GS changes from biopsy to prostatectomy. The gray box in the 1–4 core group is much larger than that in the 5–6 core group, indicating that the GS changes from biopsy to prostatectomy are significantly greater in the 1–4 core group than the 5–6 core group (*p =* 0.027).

Biopsy GS was significantly different between the 1–4 core and 5–6 core groups before (*p =* 0.008) and after (*p =* 0.006) matching even though RP GS was not statistically different before (*p =* 0.207) or after (*p =* 0.092) matching (**Table 3**). The changes from biopsy to RP GS were significantly different between the matched groups (*p =* 0.027). The GS changes were more widely distributed in the 1–4 core group than the 5–6 core group (**Figure 6**).

International Society of Urological Pathology (ISUP) upgrade of the 1–4 core and 5–6 core groups was 36.5% (95/260) and 25.0% (36/144) (*p =* 0.020), respectively. In ISUP grading, 15.8% (41/260) of the 1–4 core and 8.3%(12/144) of the 5–6 core group was upgraded more than 1 point (*p =* 0.034). In PI-RADS 5 lesion, 43.8% of patients in the 1–4 core group developed a GS upgrade and 22.2% in the 5–6 core group detected a GS upgrade (**Table 4**).

**Table 4:** Sub-analysis of Gleason score upgrade.

	Gleason score upgrade		*p*-value
	1–4 core group (107/260, 41.15%)	5–6 core group (38/144, 26.39%)	
ISUP upgrade (%)	95 (36.54)	36 (25.00)	0.020
ISUP upgrade (>1 point) (%)	41 (15.77)	12 (8.33)	0.034
PI-RADS 3 (%)	19/29 (65.52)	5/14 (35.71)	0.102
PI-RADS 4 (%)	49/142 (34.51)	23/85 (27.06)	0.302
PI-RADS 5 (%)	39/89 (43.82)	10/45 (22.22)	0.015
Image fusion biopsy (%)	66/154 (42.86)	0/2 (0)	0.509
Cognitive biopsy (%)	41/106 (38.68)	38/142 (26.76)	0.054

ISUP, International Society of Urological Pathology; PI-RADS, prostate imaging and reporting and data system.

The complication rate of the 1–4 core group was slightly higher than that of the 5–6 core group, but there was no statistical difference between the groups before and after matching (**Table 3**).

## Discussion

This study showed that 5 or more target cores in an index tumor can minimize underestimation of GS score compared with GS in an RP specimen. Moreover, targeting the periphery as well as center of an index tumor allowed detection of additional higher GS and reduced GS discrepancy between biopsy and RP.

With introduction and development of mpMRI to diagnose PCa, a target biopsy approach is being implemented widely due to its effectiveness and accuracy. However, the number of target cores varies across institutions and can vary within an institution, depending on the clinician (25). The American Urological Association recommends a target biopsy of 2 cores or more (26), and the PRECISION trial recommended a target biopsy of 4 cores for PCa (27). Recently, Tu et al. reported that 3–4 cores were better than 1–2 cores, and more than 4 cores showed a better diagnosis of significant PCa than did 4 or fewer cores (28). However, increasing the number of target biopsy cores can increase patient discomfort as well as the potential risk of complications, such as bleeding and acute prostatitis (29). Therefore, many studies have reported the number of target cores to maximize the diagnostic rate without increasing complications.

Previous studies have focused on the saturated target cores for detecting significant PCa (26–28). Diagnosis of significant PCa is important, but accurate diagnosis of GS is equally important in biopsy, as tumor burden and aggressiveness of PCa can be evaluated based on the biopsy results and can help determine a treatment plan and allow prognosis to be assessed. Previous studies reported that high preoperative PSA, high PSAD, obesity, and old age were risk factors for GS upgrade (11, 30, 31), while underlying disease, familial history, and clinical stage were not significantly associated with GS upgrade (32, 33). Our study also showed that PI-RAD classification, clinical stage, number of target cores, biopsy GS, and percentage of tumors in a biopsy core influenced GS upgrade. Therefore, the GS upgrade rate was based on the number of target cores in our study, but a propensity score matching was also used with PSA level, and many clinic-pathologic factors did not differ between the matched groups. As a result, we were able to control possible confounders that can influence GS upgrade.

Although 5ARI increased risk of high-grade PCa (34), in this study, 5ARI did not affect GS upgrading. Among the patients enrolled in this study, only 9 patients (7 in the 1–4 core vs. 2 in the 5–6 core group) had a history of surgery for benign prostatic hyperplasia (BPH) in the past. With a very small number of patients, it was impossible to evaluate the effect of BPH surgery such as TURP on the biopsy method for optimal PCa detection.

In our study, more than 36% of GS upgrades were observed for those with 4 cores or fewer, 24.39% for 5 cores, and 26.51% for 6 cores. It has been previously reported that biopsy of 5 cores or more is recommended for detecting significant PCa (17, 35, 36). These investigations did not determine where target cores were sampled in an index lesion. Therefore, to assess the target location, we obtained 3 cores in the center and the other cores in the periphery of an index lesion. This finding indicates that targeting only the center of an index lesion can miss higher GS for PCa. Additional sampling of the periphery of an index lesion helps to reduce underestimation of GS. The results of previous studies and of this study indicate that the optimal number of target cores in an index lesion is at least 5.

Recently, however, we sampled only two or three cores from PI-RADS 5 with aggressive findings such as extra-capsular extension or seminal vesicle invasion or in patients that were not able to stop medications such as aspirin or anticoagulant therapy. Consequently, our strategy applying target biopsy is not currently recommended in such clinical settings.

Our cases were matched 2:1 but not 1:1 to reduce significant loss of subjects in the 1–4 core group. The number of this group was almost two times greater than that of the 5–6 core group. Several studies have reported that variable ratio matching mostly outperforms 1:1 ratio matching (37, 38).

Downsizing tumor can be related to MRI limitations. That could be also called “MRI undersizing”: tumor located at margins of the MR lesion are not visible at MRI (39). That is related to tumor heterogeneity in terms of not-round tumor shape and volume other than histologic sub-type and grading (39). Combining these variables, we can imagine how many possible variations could influence final tumor extension and exact location of the most aggressive tumor.

In order to reduce these variables and technical software fusion collimation error: 2-5 mm or cognitive error other than Operator targeting failure (software or cognitive), in 2016, originally Galosi et al. reported the zonal saturation biopsy that overcome bias related to the most important variables (MRI interpretation, collimation, and operator) (40). Recently, in concordance with observation of this study and the former, Tschirdewahn et al. showed that target saturation biopsy detected significantly higher grade disease than Target Biopsy and extended Systematic Biopsy (41).

In the present study, among target biopsy cores, the ratio of the positive cores was 79.6% in the 1–4 core group and 80.7% in the 5–6 core group. The median per-patient percentage of malignant tissue within biopsy cores was 50% (IQR: 22.5–70%) in the 1–4 core group and 60% (IQR: 40–72.5%) in the 5–6 core group. Compared with previous studies, accuracy of the biopsy in this study is reliable (42).

This study had some limitations. First, as it was a retrospective study, the possibility of selection bias cannot be excluded. The cohort was restricted to those treated with prostatectomy, who tend to have smaller tumors at lower stage. Thus, the biopsy sampling strategy may not need to be as aggressive in patients who have larger, more advanced tumors. Second, the optimal number of systematic biopsies was not determined, and systematic biopsy could detect additional significant PCa. Third, there was a lack of analysis according to risk category rather than Gleason score. Underestimation of risk category (i.e., low-, intermediate-, or high-risk histology) rather than simply Gleason score is of more clinical importance and analyzing along those lines would be useful. Montironi et al. (43) showed two relevant issues: the first, ISUP grading is the preferred and easy method to be used compared to Gleason score to evaluate grade difference between biopsy and pathology; the second is that more than 1 ISUP point has a profound impact on disease management rather than 1 point upgrading. In this study, in the 5–6 core group, ISUP upgrade was significantly lower than the 1–4 core group. However, clinical differences, such as biochemical recurrence and adjuvant treatment, between two groups could not be identified according this classification, so further studies will be needed. Fourth, the study population was not uniformly composed. Two radiologists used different techniques: one used cognitive biopsy in all cases and the other did image fusion biopsy in almost all cases.

## Conclusion

Increasing the number of target cores is useful for minimizing GS underestimation without increasing the complication rate. Based on this analysis, the number of target cores should be five or more. Sampling the periphery as well as center of an index lesion is a key technical step for obtaining higher GS PCa.

## Data Availability Statement

The original contributions presented in the study are included in the article/supplementary material. Further inquiries can be directed to the corresponding author.

## Ethics Statement

The studies involving human participants were reviewed and approved by Samsung Medical Center. Written informed consent for participation was not required for this study in accordance with the national legislation and the institutional requirements.

## Author Contributions

Conceptualization: All authors. Methodology: All authors. Software: JC. Validation: BP. Formal analysis: JC. Investigation: BP. Resources: BP. Data curation: BP. Writing—original draft preparation: JC and BP. Writing—review and editing: All authors. Visualization: BP. Supervision: BP. Project administration: BP. Funding acquisition: none. All authors contributed to the article and approved the submitted version.

## Conflict of Interest

The authors declare that the research was conducted in the absence of any commercial or financial relationships that could be construed as a potential conflict of interest.

## Publisher’s Note

All claims expressed in this article are solely those of the authors and do not necessarily represent those of their affiliated organizations, or those of the publisher, the editors and the reviewers. Any product that may be evaluated in this article, or claim that may be made by its manufacturer, is not guaranteed or endorsed by the publisher.
